# Artificial intelligence in carotid research: a 25-year bibliometric analysis of global trends and future directions

**DOI:** 10.3389/frai.2026.1716935

**Published:** 2026-05-19

**Authors:** Shi-Chao Zhu, Jia-Xin Dong, Yu-Dan Wu, Yue Gao, Rui Gao, Yong-Hui Yuan, Shuang Wang, Guang-Wei Zhang

**Affiliations:** 1 Department of General Practice, The First Hospital of China Medical University, Shenyang, China; 2 School of Nursing, Fudan University, Shanghai, China; 3 Cancer Hospital of Dalian University of Technology, Shenyang, China; 4 Liaoning Cancer Hospital & Institute, Clinical Research Center for Malignant Tumor of Liaoning Province, Cancer Hospital of China Medical University, Shenyang, China; 5 Department of Smart Hospital Management, The First Hospital of China Medical University, Shenyang, China; 6 The Internet Hospital Branch of the Chinese Research Hospital Association, Beijing, China

**Keywords:** artificial intelligence, bibliometrics, carotid, CiteSpace, visual analysis

## Abstract

**Background:**

Artificial intelligence (AI) technology advancements have revolutionized carotid artery research, driving innovations in diagnostics and treatment. The exponential growth of studies underscores the critical need for systematic analysis to ensure clinical relevance and algorithmic robustness.

**Objective:**

To systematically evaluate the status of AI applications in carotid artery research, identify emerging themes and track development trends, and establish a guiding framework for future research.

**Methods:**

A retrospective bibliometric analysis of Web of Science Core Collection data, Scopus, and PubMed databases from 2000 to 2025 was performed, focusing on title/abstract/keyword queries related to AI-driven carotid artery research. Network visualizations and structural analyses generated by CiteSpace reveal evolving thematic clusters and emerging frontiers in this interdisciplinary field.

**Results:**

A total of 1,220 relevant publications were identified in this study, with an overall increasing trend in publication volume. China and the United States emerged as the primary contributing countries. Research mainly focuses on early, accurate diagnosis, monitoring, individualized intervention of carotid artery disease, imaging omics analysis, and advanced physiological and pathological analysis. Recent progress is characterized by big data, multi-omics, multi-modality, and multi-scale integration, emphasizing precision medicine and full-process evaluation of disease management, marking a major shift in carotid research toward a data-driven approach.

**Conclusion:**

This bibliometric study employs visualization techniques to delineate AI’s transformative role in carotid artery research, demonstrating its potential to redefine research paradigms and drive sustained advancements through advanced data analytics.

## 1 Introduction

The carotid artery is regarded as one of the key research subjects in cardiovascular and cerebrovascular diseases. To achieve accurate diagnosis, risk assessment, and formulation of appropriate treatment plans, an increasing number of studies have focused on imaging analysis of the carotid artery, its anatomy, hemodynamics, and the underlying pathophysiological mechanisms in fundamental physiological research (Saba et al., 2018; Liu et al., 2022; Meloni et al., 2024; Ahmed et al., 2024; Mac Grory et al., 2020; Raju et al., 2020; Kigka et al., 2021). Through an in-depth understanding of the carotid artery to identify early features, mechanisms, and processes of cerebrovascular diseases, effective means of prevention, treatment, and intervention can be provided. This, in turn, enhances clinical treatment outcomes and improves the quality of life for patients (Willeit et al., 2020; Bonati et al., 2022). However, traditional carotid artery research faces several challenges due to the limitations of its research methods. For example, in carotid artery pathology studies, histological analysis relies on samples obtained from patients, which are costly, time-consuming, and difficult to capture the three-dimensional structure and dynamic changes of the entire plaque. On the other hand, imaging examinations struggle to sensitively detect early or mild carotid stenosis, hemodynamic changes, and microlesions. Moreover, these methods are susceptible to factors such as operator skill, equipment requirements, and patient positioning, which limit their accuracy and generalizability. The consistency of diagnostic results is also challenging, and some examinations (e.g., angiography) even carry invasive risks (Del Brutto et al., 2020).

With the rapid development of artificial intelligence (AI), its application in medicine has become a key factor in driving improvements in diagnostic and treatment efficiency (Hamet and Tremblay, 2017; Martelli et al., 2024). Breakthroughs in AI, particularly in areas such as medical imaging, machine learning, and data mining, are providing new perspectives and methods for the diagnosis, prediction, and treatment of carotid artery diseases (Abd-Ellah et al., 2021; Stoitsis et al., 2006; Saba et al., 2019; Albertini et al., 2024; Amritphale et al., 2021). In addition, AI technology has opened new directions for the application of carotid artery research, such as the use of carotid pressure waveforms to monitor blood pressure (Zhao et al., 2023), estimate arterial compliance (Bikia et al., 2021), and assess vascular aging (Jin et al., 2021). Some studies have also used AI to recognize carotid artery sounds for individual biometric identification and verification (Salvi et al., 2021). These applications can generally be divided into experimental and clinical domains. Experimental applications primarily focus on methodological development, including signal processing, image segmentation, and predictive modeling, whereas clinical applications emphasize diagnostic assistance, risk stratification, disease monitoring, and treatment decision support in patients with carotid artery disease. The full application of AI has the potential to revolutionize current diagnostic and treatment models, playing a crucial role in research related to carotid artery disease diagnosis, disease prediction, personalized treatment, real-time monitoring, and fundamental research. As AI continues to be integrated into carotid artery research, it is expected that more studies will make progress in developing more precise, efficient, and personalized diagnostic and treatment plans, offering new approaches and solutions for managing cardiovascular and cerebrovascular events. The accumulation of relevant literature is significantly altering our approach to the research, diagnosis, and management of carotid artery diseases.

However, despite the expanding body of research, systematic analysis of trends and cutting-edge topics in this field remains relatively scarce. The lack of understanding of the overall development trends in this area limits our deeper comprehension of AI-based carotid artery research. Bibliometrics is a branch of informatics that emphasizes the use of objective quantitative analysis methods to examine bibliographic data. It is used to reveal trends in academic development, research hotspots, and patterns of scientific collaboration, among other aspects (Pritchard, 1969; Liu et al., 2024). This method can measure the profile distribution, relationships, and clustering within a research field. It can also be used to compare the contributions of authors, institutions, countries, and journals (Ninkov et al., 2022). Given its ability to provide insights into resource management, research collaboration, current status, development trends, and journal impact, bibliometrics is highly suitable for offering a comprehensive overview of AI-based research in the field of carotid artery studies.

Although bibliometric methods have been widely applied in many research areas, systematic bibliometric analyses focusing specifically on AI applications in carotid artery research remain limited. Therefore, this study conducted a comprehensive bibliometric analysis to explore global publication trends, core research hotspots, and emerging research frontiers in this field. By analyzing countries, authors, journals, institutions, and keywords, the study offers a comprehensive review of the application and development of AI in this field. Based on the current state of scientific progress, this study aims to provide scientific evidence and guidance for future research directions and practical applications of AI in carotid artery studies.

## 2 Method

### 2.1 Data source and literature selection

**Figure 1:**
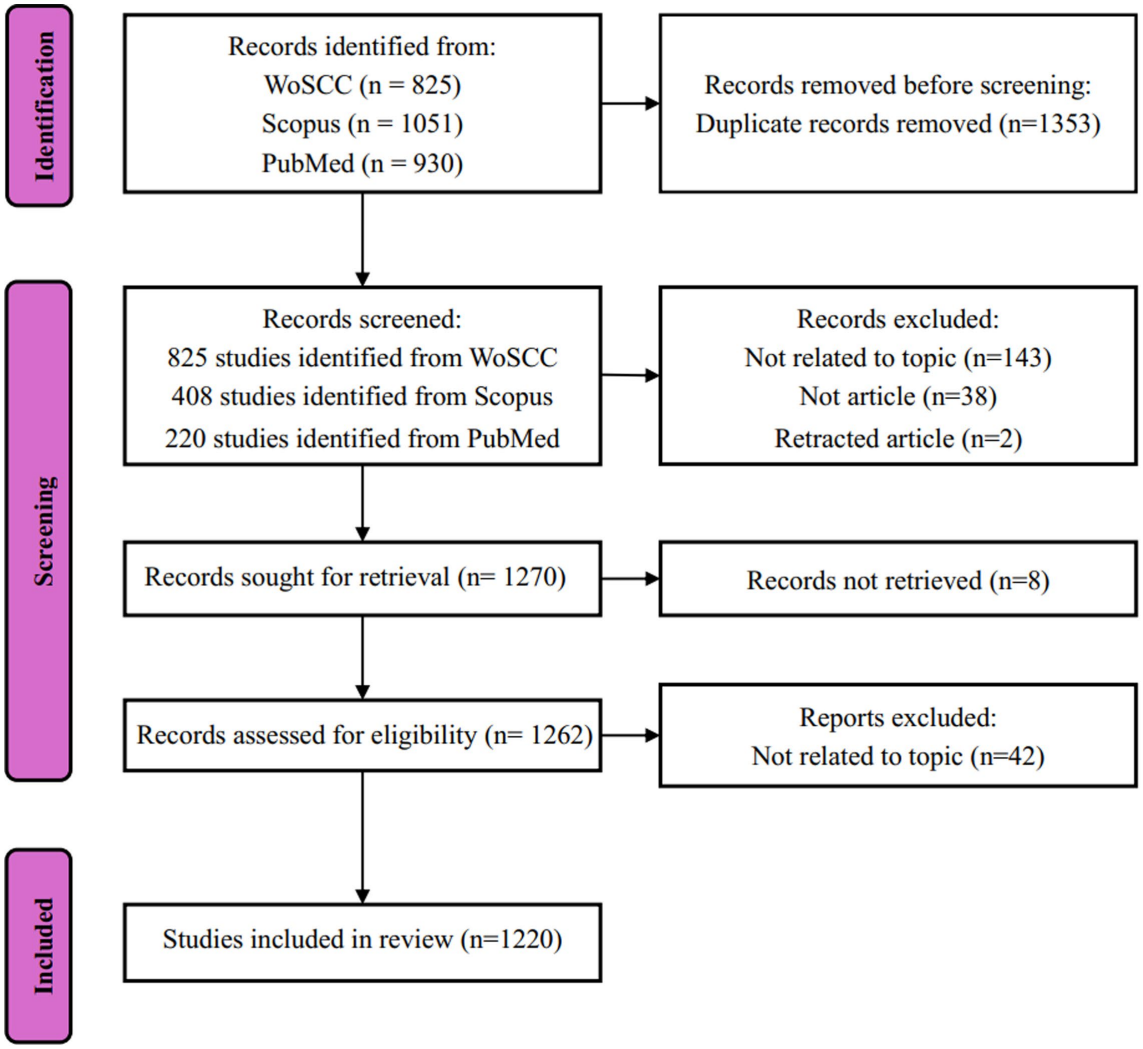
Overview of the literature collection and the exclusion criteria.

This study conducted literature searches using the Web of Science Core Collection (WoSCC), Scopus, and PubMed databases. WoSCC was selected for its recognized authority and comprehensive coverage across disciplines, establishing it as a predominant resource for bibliometric analyses with robust citation data capabilities, as documented in prior research (Liu et al., 2024). Scopus was employed as a major global abstract and citation database, valued for its reliable bibliometric indicators that support research assessment and institutional rankings (Baas et al., 2020). PubMed served as the specialized resource for biomedical literature, ensuring retrieval of high-quality content in this domain (Ossom Williamson and Minter, 2019), though it does not provide detailed citation analysis features comparable to the other two databases. This study adopted a bibliometric analysis design rather than a conventional intervention-based systematic review. Therefore, the scope of included studies was not limited to specific interventions or outcomes, but instead encompassed methodological, technical, and clinical research related to artificial intelligence applications in carotid artery studies. The selection criteria were as follows: (i) articles published before December 31, 2025; (ii) related to AI and carotid artery; (iii) published in English; (iv) peer-reviewed articles. To remove duplicate records, the “Deduplication” function of CiteSpace 6.4. R1 software was used for initial cleaning. Applying an English language restriction is to ensure the quality and standardization of the data, because English is still the main medium for international scientific communication and inclusion in the WoS. All retrieved records were imported into reference management software for data cleaning and deduplication. Two reviewers independently conducted title and abstract screening, followed by full-text assessment when necessary. Discrepancies were resolved through discussion to reach consensus. This dual-review process ensured consistency and minimized selection bias during study inclusion. The literature search was completed on January 2, 2026, and after screening, a total of 1,220 records were included in this study (Figure 1). The detailed search strategy is shown in Table 1.

**Table 1:** Main parameter settings used in CiteSpace analysis.

Category	Parameter	Setting
Software	Software version	CiteSpace (Version 6.4.1)
Time slicing	Time span	2000–2025
Time slicing	Years per slice	1 year
Node selection	Selection criteria	Top 50 per slice
Node types	Types analyzed	Author, Institution, Country, Keyword, Reference, Cited Author, Cited Journal
Network pruning	Pruning method	Pathfinder
Network pruning	Additional pruning	Pruning sliced networks
Clustering	Clustering algorithm	Log-likelihood ratio (LLR)
Cluster evaluation	Quality indicators	Modularity Q and Silhouette score
Centrality	Network metric	Betweenness centrality
Burst detection	Algorithm	Kleinberg burst detection
Visualization	Network views	Cluster view, Timeline view and Timezone view

### 2.2 Visual analysis software

CiteSpace (v.6.4.R1 Advanced) software is a Java application developed by Dr. Chaomei Chen from Drexel University. CiteSpace uses set theory-based data normalization methods to measure the similarity of knowledge units, aiming to understand the development process and trends in a given field (Chen, 2006). The software supports data import, node selection, parameter configuration, and other functionalities, which help in identifying research hotspots, trends, and key nodes. Key parameters, including time slicing, node types, and pruning strategies, were selected based on commonly adopted settings in previous bibliometric studies. Except for the parameters explicitly described in the Methods section, all remaining settings followed the default configurations of CiteSpace. To enhance transparency and reproducibility, a summary of the main CiteSpace parameter settings used in this study is provided in Supplementary material S1.

### 2.3 Data analysis

We exported complete literature data from the Web of Science core database, including Full Record and Cited References as plain text, and then imported them into CiteSpace for further analysis. This dataset was used to create a Bibliometric Map to reveal the evolution and flow of scientific knowledge, and by using literature data from 2000 to 2025, a time slice analysis of knowledge was performed to show the trends in research fields and changes in knowledge structure between different years, so as to reveal information such as changing trends, evolutionary processes, and scientific research hotspots within disciplines or research fields. In addition, we calculated the centrality index using the co-citation relationship in CiteSpace to evaluate the importance of nodes in the research network. The higher the centrality index, the greater the influence on information dissemination and research influence. The centrality index was calculated using the co-citation relationship in CiteSpace. We adopted a strategy combining the pathfinder and the pruning sliced networks to more efficiently extract the core relationships between documents. This will help reveal potential important connections between documents and also improve the clarity and interpretability of visual analysis. The burst detection algorithm proposed by Kleinberg, J was used to identify emerging research topics to enhance our understanding of the latest developments and future directions in the field (Kleinberg, 2003).

## 3 Result

### 3.1 Distribution of article publication years

**Figure 2:**
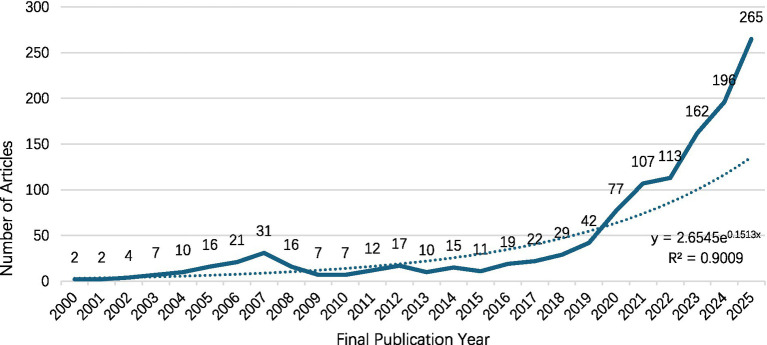
The global trend of AI-based carotid research publication from 2000 to 2025. The dotted line represents the fitted exponential growth trend of annual publication counts (*R*^2^ = 0.9009).

From 2000 to 2025, the number of published papers in the field of AI and carotid artery research generally showed a fluctuating upward trend and generally followed an exponential growth pattern (Figure 2). The number of publications fluctuated at relatively low levels for over a decade until 2016, when annual publication numbers began to surge, indicating a significant increase in research interest in this field, suggesting that the field continues to make breakthroughs and maintain an active research momentum (Table 2).

**Table 2:** Search query syntax by components.

Component	Advanced search query syntax		
	Web of science	Scopus	PubMed
#1 Artificial Intelligence	TS = (“Machine Learning” OR “Artificial Intelligence” OR “Deep learning” OR “Supervised model*” OR “Unsupervised model*” OR “Supervised algorithm*” OR “Unsupervised algorithm*” OR “supervised learning” OR “Unsupervised learning” OR “Semi-supervised learning” OR “Reinforcement learning” OR “AI algorithm*” OR “DL algorithm*” OR “ML algorithm*” OR “AI model*” OR “DL model*” OR “ML model*” OR “neural network*” OR “Large Language Model*” OR “Generative Pre-Trained Transformer” OR “Radiomic*” OR “Ultrasomic*”)	(KEY (“machine learning” OR “artificial intelligence” OR “deep learning” OR “supervised learning” OR “unsupervised learning” OR “semi-supervised learning” OR “reinforcement learning” OR “neural network” OR “neural networks” OR “large language model” OR “generative ai” OR “radiomic” OR “radiomics” OR “ultrasomic” OR “ultrasomics”) OR TITLE-ABS (“supervised model*” OR “unsupervised model*” OR “supervised algorithm*” OR “unsupervised algorithm*” OR “ai algorithm*” OR “dl algorithm*” OR “ml algorithm*” OR “ai model*” OR “dl model*” OR “ml model*” OR “transformer model” OR “gpt”))	“Machine Learning”[Title/Abstract] OR “Artificial Intelligence”[Title/Abstract] OR “deep learning”[Title/Abstract] OR “supervised model*”[Title/Abstract] OR “unsupervised model*”[Title/Abstract] OR “supervised algorithm*”[Title/Abstract] OR “unsupervised algorithm*”[Title/Abstract] OR “supervised learning”[Title/Abstract] OR “Unsupervised learning”[Title/Abstract] OR “Semi-supervised learning”[Title/Abstract] OR “Reinforcement learning”[Title/Abstract] OR “ai algorithm*”[Title/Abstract] OR “dl algorithm*”[Title/Abstract] OR “ml algorithm*”[Title/Abstract] OR “ai model*”[Title/Abstract] OR “dl model*”[Title/Abstract] OR “ml model*”[Title/Abstract] OR “neural network*”[Title/Abstract] OR “large language model*”[Title/Abstract] OR “Generative Pre-Trained Transformer”[Title/Abstract] OR “radiomic*”[Title/Abstract] OR “ultrasomic*”[Title/Abstract] OR “Machine Learning”[MeSH Terms] OR “Artificial Intelligence”[MeSH Terms] OR “neural networks, computer”[MeSH Terms] OR “deep learning”[MeSH Terms]
#2 Carotid Artery	TS = (“Carotid Arter*” OR “Caroti*” OR “neck arter*”)	(KEY (“carotid artery” OR “carotid arteries”) OR TITLE-ABS (“carotid arter*” OR “caroti*” OR “neck arter*”))	“carotid arter*”[Title/Abstract] OR “caroti*”[Title/Abstract] OR “neck arter*”[Title/Abstract] OR “Carotid Arteries”[MeSH Terms]
#3 animal research	TS = (animal OR mouse OR mice OR rat OR rats OR rabbit* OR dog* OR pig* OR swine* OR cow* OR cattle* OR Sheep* OR goat* OR murine*)	(TITLE-ABS-KEY (animal OR mouse OR mice OR rat OR rats OR rabbit* OR dog* OR pig OR swine OR cow OR cattle OR Sheep* OR goat* OR murine*)	“Animals”[MeSH Terms] NOT “Humans”[MeSH Terms]
#4 Limited to Article			“journal article”[Publication Type] NOT (“review”[Publication Type] OR “systematic review”[Publication Type] OR “meta-analysis”[Publication Type])
#5	#1 AND #2 NOT #3 Filters: Article, English, from 2000–2025	#1 AND #2 AND NOT #3 Filters: Article, English, from 2000–2025	(#1 AND #2) NOT #3 AND #4 Filters: English, from 2000–2025

### 3.2 Country/region analysis

The top ten countries in terms of the number of published papers are China, the United States, Italy, Canada, and United Kingdom, India, Japan, Germany, Turkey, and Netherlands (Table 3). China and the United States exhibited a pronounced tiered dominance in publication output, indicating that both countries are at the forefront of global research on AI–assisted carotid artery studies and play a leading role in the development of this field. China ranked first with 386 publications, reflecting rapid growth in research investment and output; however, its relatively low centrality index (0.06) suggests that its role as a hub within the international collaboration network still has room for improvement. In contrast, the United States ranked second with 312 publications but showed a markedly higher centrality (0.24), highlighting its core position in global research collaboration and stronger academic influence. The discrepancy between publication volume and network centrality between the two countries reflects distinct development patterns, with China emphasizing rapid expansion of research output, whereas the United States maintains advantages in international collaboration and academic leadership. Other countries also maintained a high level of research activity, indicating that the field has developed into a multinational research landscape.

**Table 3:** Top 10 countries/regions contributing to publications.

Rank	Country/region	Centrality	Frequency
1	Peoples R China	0.06	386
2	USA	0.24	312
3	Canada	0.04	96
4	Italy	0.07	92
5	United Kingdom	0.09	90
6	India	0.01	74
7	Germany	0.05	58
8	Japan	0.03	54
8	Netherlands	0.03	54
10	Turkey	0.00	49

**Figure 3:**
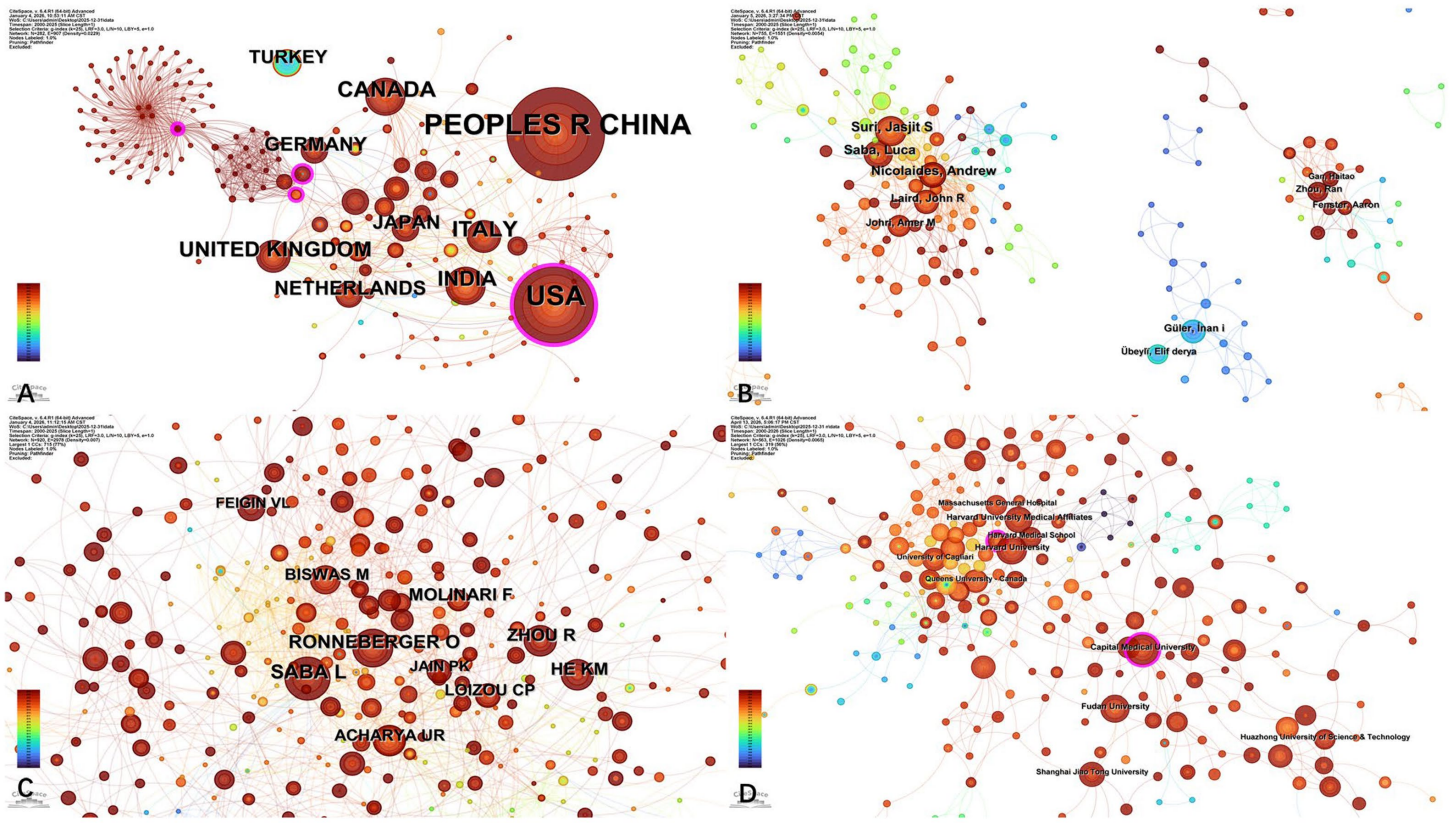
Distribution of countries/regions, authors and cited authors, institutions. **(A)** Map of country/regional cooperation network analysis. **(B)** Map of author cooperation network analysis. **(C)** Map of cited author analysis. **(D)** Map of institution cooperation network analysis.

The research network map of various countries (Figure 3A) reveals a relatively loose cooperation model (*n* = 282, E = 907, density = 0.0229), which shows that this research field has a certain appeal and breadth internationally. However, despite the participation of more countries, there are relatively few cooperative relationships between countries, and the cooperative relationships are relatively scattered. Because there are relatively few cooperative relationships between countries, there may be an imbalance in cooperation, which is manifested in that some countries may have closer cooperation, while other countries have lower participation. A few scientific research powers (such as China, the United States, England, etc.) dominate this field and lead international cooperation and research directions. This pattern may reflect fragmented research efforts, discipline-specific funding mechanisms, and limited cross-national data-sharing frameworks, which collectively constrain large-scale international collaboration despite high research productivity. But it also means that the field has the potential to promote innovation and development in scientific research by expanding the depth and breadth of collaborative networks.

### 3.3 Author distribution

A total of 1,030 authors have contributed to the field, and Table 4 lists the top 10 authors with the most published articles. Suri, Jasjit S of University of Idaho, USA, ranked first with 51 articles. They were followed by Nicolaides, Andrew of The Chinese University of Hong Kong, China, and Saba, Luca of University of Cagliari, Italy, with 38 and 30 articles, respectively. This reflects the significant academic contributions and leading position of these scholars in the field, while also highlighting the trend of diversification in research activities within this area. Table 5 further summarizes the top 5 most cited authors, offering additional insight into author-level academic impact.

**Table 4:** Top 10 most published authors.

Rank	Author	Country/region	Institution	Article counts
1	Suri, Jasjit S	USA	University of Idaho	51
2	Nicolaides, Andrew	Peoples R China	The Chinese University of Hong Kong, CUHK	50
3	Saba, Luca	Italy	University of Cagliari	48
4	Laird, John R	USA	University of California, Davis	27
5	Güler, İnan i	Turkey	Gazi University	20
6	Johri, Amer M	Canada	Queen’s University	19
7	Übeylï, Elif derya	Turkey	TOBB University of Economics and Technology	16
8	Fenster, Aaron	Canada	Robarts Research Institute	15
9	Zhou, Ran	Peoples R China	Hubei University of Technology	14
10	Gan, Haitao	Peoples R China	Hubei University of Technology	11

**Table 5:** Top 5 authors with the most citations.

Rank	Author	Country/region	Institution	Article counts
1	Saba, Luca	Italy	University of Cagliari	149
2	Ronneberger O	Germany	University of Freiburg	101
3	He KM	USA	Massachusetts Institute of Technology	70
4	Zhou R	Peoples R China	Hubei University of Technology	69
5	Molinari F	Italy	University of Pavia	67

The analysis of the author collaboration network (Figure 3B) and the cited author network (Figure 3C) collectively reveals the fragmented nature of academic exchanges in this field. The collaboration network (*n* = 755, E = 1,551, density = 0.0054) indicates sparse collaborative relationships among authors, with only a few core authors dominating the network while the majority of researchers remain in peripheral collaborative roles. The overall low network density suggests that widespread and tightly knit interactive patterns of collaboration have yet to emerge. This fragmented collaboration structure may partly reflect the interdisciplinary nature of AI-based carotid artery research, which involves separate research communities in clinical medicine, medical imaging, and computer science. In addition, differences in funding mechanisms and data accessibility may further limit the formation of large-scale collaborative networks. Similarly, the cited author network (*n* = 1,099, E = 3,324, density = 0.0055) also exhibits dispersed citation relationships. Despite the presence of some highly influential scholars, the pathways for the transmission of academic ideas are relatively narrow, with insufficient cross-disciplinary or cross-regional depth in citations. This structure may hinder large-scale innovation and multicenter validation, which are essential for translating AI-based carotid research into robust clinical applications. Moving forward, it is essential to strengthen interdisciplinary collaboration, promote international joint research, and establish more open academic exchange platforms to foster interactions between core authors and peripheral researchers. Additionally, encouraging diversified citation practices can enhance network density, thereby improving academic synergy and innovation potential in the field.

### 3.4 Institutional analysis

In the institutional ranking (Table 6), the top contributing institutions were predominantly from China and the United States. Capital Medical University ranked first with 33 publications and demonstrated the highest betweenness centrality (0.14), suggesting its pivotal role in both research output and institutional collaboration. Harvard University and Harvard University Medical Affiliates ranked second and third with 32 and 29 publications, respectively, highlighting the prominent role of U.S. institutions in this field. The institutional research network (Figure 3D) exhibits a low connection density (*n* = 563, E = 1,026, density = 0.0065), indicating limited collaboration intensity among academic institutions. Although the network has a broad coverage, reflecting the global nature of research, collaborations are predominantly concentrated among a few institutions, with others having lower participation. This distribution pattern may reflect regional or disciplinary barriers, and interdisciplinary collaboration remains in its exploratory stages. Strengthening institutional cooperation, particularly across borders and disciplines, and integrating dispersed academic resources are key directions for optimizing the structure and efficiency of the research network.

**Table 6:** Top 10 institutions with the most publications.

Rank	Institution	Centrality	Frequency
1	Capital Medical University	0.14	33
2	Harvard University	0.02	32
3	Harvard University Medical Affiliates	0.01	29
4	Fudan University	0.03	29
5	Shanghai Jiao Tong University	0.04	23
6	University of Cagliari	0.01	22
6	Queens University - Canada	0.03	22
8	Harvard Medical School	0.04	21
9	Massachusetts General Hospital	0.04	19
10	Huazhong University of Science & Technology	0.00	19

### 3.5 Journal analysis and most cited articles

Citation analysis (Table 7) shows that *STROKE* (461 citations), *CIRCULATION* (335 citations), and *RADIOLOGY* (223 citations) are the most cited journals in AI-assisted carotid artery research, reflecting their central role in disseminating influential findings. High-impact journals such as *IEEE T MED IMAGING*, *J AM COLL CARDIOL*, and *LANCET* further highlight the field’s clinical and methodological significance, with *LANCET*’s exceptional impact factor (88.5) indicating its authoritative academic status. The 10 most cited articles (Table 8) reveal core research themes and key contributors, exemplified by Zhang RY’s review on MRI-based radiomics and machine learning, which received the highest citations (*n* = 32), illustrating the growing importance of AI-based radiomic approaches in carotid artery research.

**Table 7:** Top 10 journals with the most citation frequency.

Rank	Journal	Count	IF2024	Total publications in the journal
1	Stroke	461	8.9	42,648
2	Circulation	335	38.6	143,610
3	Radiology	223	15.2	21,410
4	IEEE T Med Imaging	207	9.8	5,745
5	J Am Coll Cardiol	207	22.3	104,118
6	Comput Biol Med	197	6.3	7,107
7	Sci Rep-UK	191	3.9	277,263
8	Ultrasound Med Biol	190	2.6	6,536
9	Lancet	177	88.5	51,374
10	Comput Meth Prog Bio	168	4.8	6,350

**Table 8:** Top 10 articles with the most citation frequency.

Rank	Title	First author	Journal	Publication year	Total citations	DOI
1	Identification of high-risk carotid plaque with MRI-based radiomics and machine learning	Zhang RY	Eur Radiol	2021	32	10.1007/s00330-020-07361-z
2	Hybrid deep learning segmentation models for atherosclerotic plaque in internal carotid artery B-mode ultrasound	Jain PK	Comput Biol Med	2021	28	10.1016/j.compbiomed.2021.104721
3	Deep learning-based carotid media-adventitia and lumen-intima boundary segmentation from three-dimensional ultrasound images	Zhou R	Med Phys	2019	27	10.1002/mp.13581
4	Two-stage artificial intelligence model for jointly measurement of atherosclerotic wall thickness and plaque burden in carotid ultrasound: A screening tool for cardiovascular/stroke risk assessment	Biswas M	Comput Biol Med	2020	21	10.1016/j.compbiomed.2020.103847
4	Imaging biomarkers of vulnerable carotid plaques for stroke risk prediction and their potential clinical implications	Saba L	Lancet Neurol	2019	21	10.1016/S1474-4422(19)30035-3
6	Automated design of deep learning methods for biomedical image segmentation	Isensee F	Nat Methods	2021	19	10.1038/s41592-020-01008-z
6	Global and regional prevalence, burden, and risk factors for carotid atherosclerosis: a systematic review, meta-analysis, and modelling study	Song PG	Lancet Glob Health	2020	19	10.1016/S2214-109X(20)30117-0
10	A low-cost machine learning-based Stroke risk stratification and its validation using ultrasonic echolucent carotid wall plaque morphology: a machine learning paradigm	Araki T	Comput Biol Med	2017	18	10.1016/j.compbiomed.2016.11.011
10	Global perspective on carotid intima-media thickness and plaque: should the current measurement guidelines be revisited?	Saba L	Int Angiol	2019	18	10.23736/S0392-9590.19.04267-6
10	A voxel-based fully convolution network and continuous max-flow for carotid vessel-wall-volume segmentation from 3D ultrasound images	Zhou R	IEEE T Med Imaging	2020	18	10.1109/TMI.2020.2975231

CNN, convolutional neural network.

### 3.6 Keyword co-occurrence analysis

**Figure 4:**
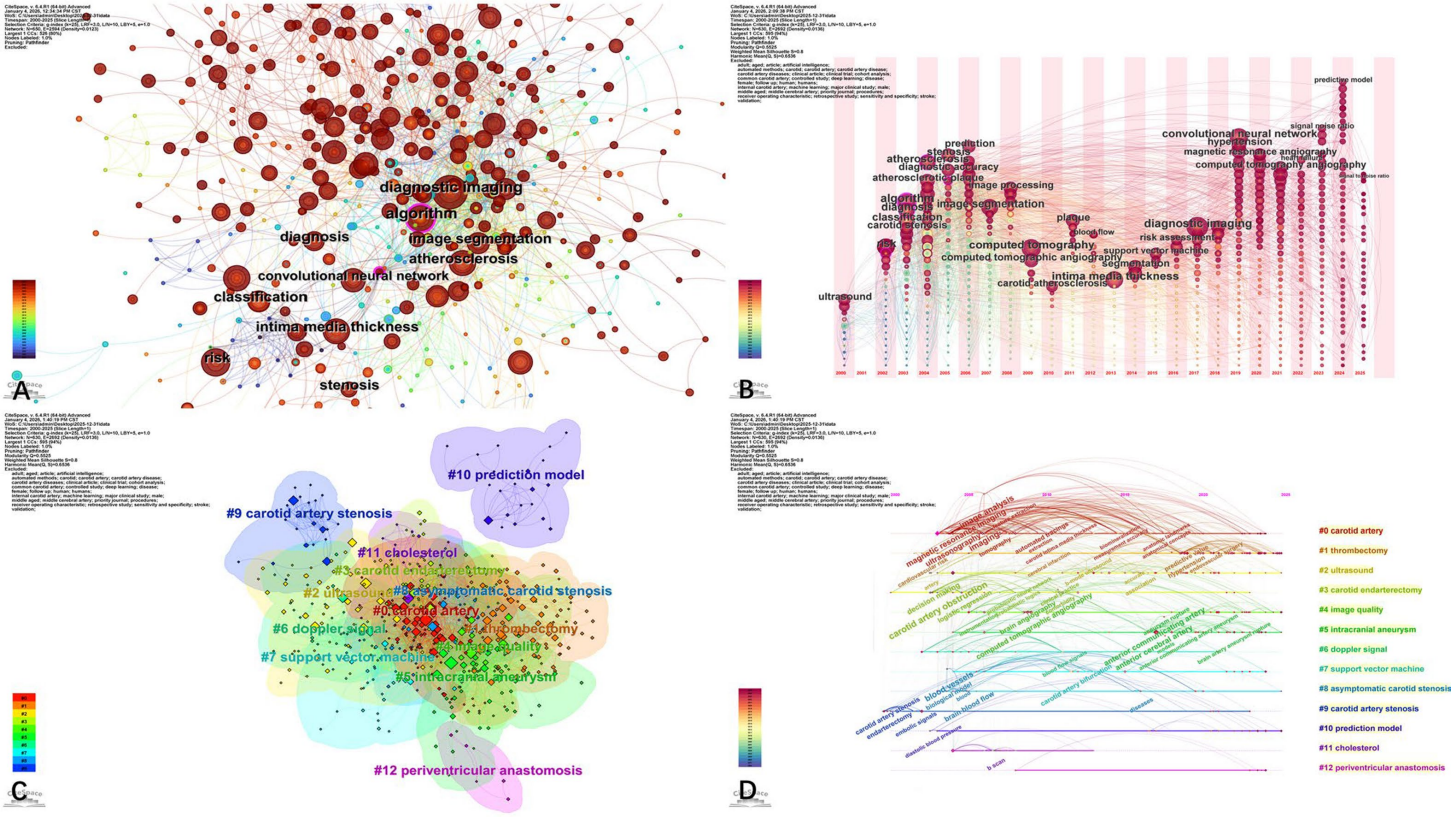
Keyword co-occurrence network analysis. **(A)** Map of keyword co-occurrence network analysis. **(B)** Keywords time zone chart. **(C)** Keywords cluster analysis map. **(D)** Keywords time line view.

We analyzed the titles and abstracts of 1,220 articles and plotted a keyword co-occurrence map (Figure 4A). The network exhibited low connection density (*n* = 650, E = 2,594, density = 0.0123), indicating a diverse set of research topics with relatively scattered interconnections, suggesting that AI-based carotid artery research is both multidisciplinary and in a stage of conceptual expansion. The 10 most frequent keywords (Table 9) were “algorithm,” “diagnostic imaging,” “intima media thickness (IMT)”, “atherosclerosis”, “convolutional neural network (CNN)”, “diagnosis”, “image segmentation”, “classification”, “risk,” and “stenosis.” These keywords indicate that AI-based carotid artery research is jointly driven by methodological development and clinically oriented objectives. High-frequency methodological terms such as “algorithm”, “CNN”, “image segmentation” and “classification” highlight the central role of computational modeling and automated image analysis, while clinically relevant keywords including “IMT”, “atherosclerosis”, “stenosis”, and “risk” reflect a strong focus on vascular pathology characterization and risk assessment. The keyword time zone view (Figure 4B) further illustrates the temporal evolution of these themes, showing an early emphasis on diagnostic imaging and disease identification, followed by increasing attention to AI-based modeling and quantitative risk evaluation, indicating a shift toward more predictive and clinically integrated research priorities.

**Table 9:** Top 10 keywords co-occurrence with the most frequency.

Rank	Keyword	Centrality	Frequency
1	algorithm	0.12	124
2	diagnostic imaging	0.01	122
3	intima media thickness	0.01	95
4	atherosclerosis	0.04	86
5	convolutional neural network	0.00	86
6	diagnosis	0.04	79
7	image segmentation	0.02	79
8	classification	0.04	78
9	risk	0.00	78
10	stenosis	0.01	65

### 3.7 Keyword co-citation clusters co-citation keyword analysis

The Log-Likelihood Ratio algorithm was employed to perform semantic clustering on 630 keywords and generate 13 clusters (Figure 4C). The Weighted Mean Silhouette Coefficient S was 0.80 (S > 0.7), indicating high cluster cohesion and clear thematic differentiation. Through semantic integration of the keyword clusters, the research landscape of AI-based carotid artery studies can be broadly structured into interrelated methodological, disease-oriented, and clinical application dimensions. At the methodological level, clusters such as “ultrasound” (#2), “doppler signal” (#6), “image quality” (#4), “support vector machine” (#7), and “prediction model” (#10) highlight a strong emphasis on AI-enabled image processing, signal analysis, and predictive modeling, particularly within ultrasound-based diagnostic frameworks. In terms of research focus, disease-related clusters including “carotid artery stenosis” (#9) and “asymptomatic carotid stenosis” (#8) are organized around the core cluster “carotid artery” (#0), underscoring its central position in both methodological development and clinical investigation. At the clinical application level, clusters associated with “thrombectomy” (#1) and “carotid endarterectomy” (#3), together with vascular risk and comorbidity-related topics such as “cholesterol” (#11) and “intracranial aneurysm” (#5), reflect the increasing integration of AI techniques into therapeutic decision-making and cerebrovascular risk assessment. The substantial overlap and interaction among clusters indicate a convergent research pattern linking algorithm development, vascular pathology, and clinical intervention, while the timeline visualization (Figure 4D) further demonstrates the dynamic evolution of these themes over time.

### 3.8 Keyword burst analysis

**Figure 5:**
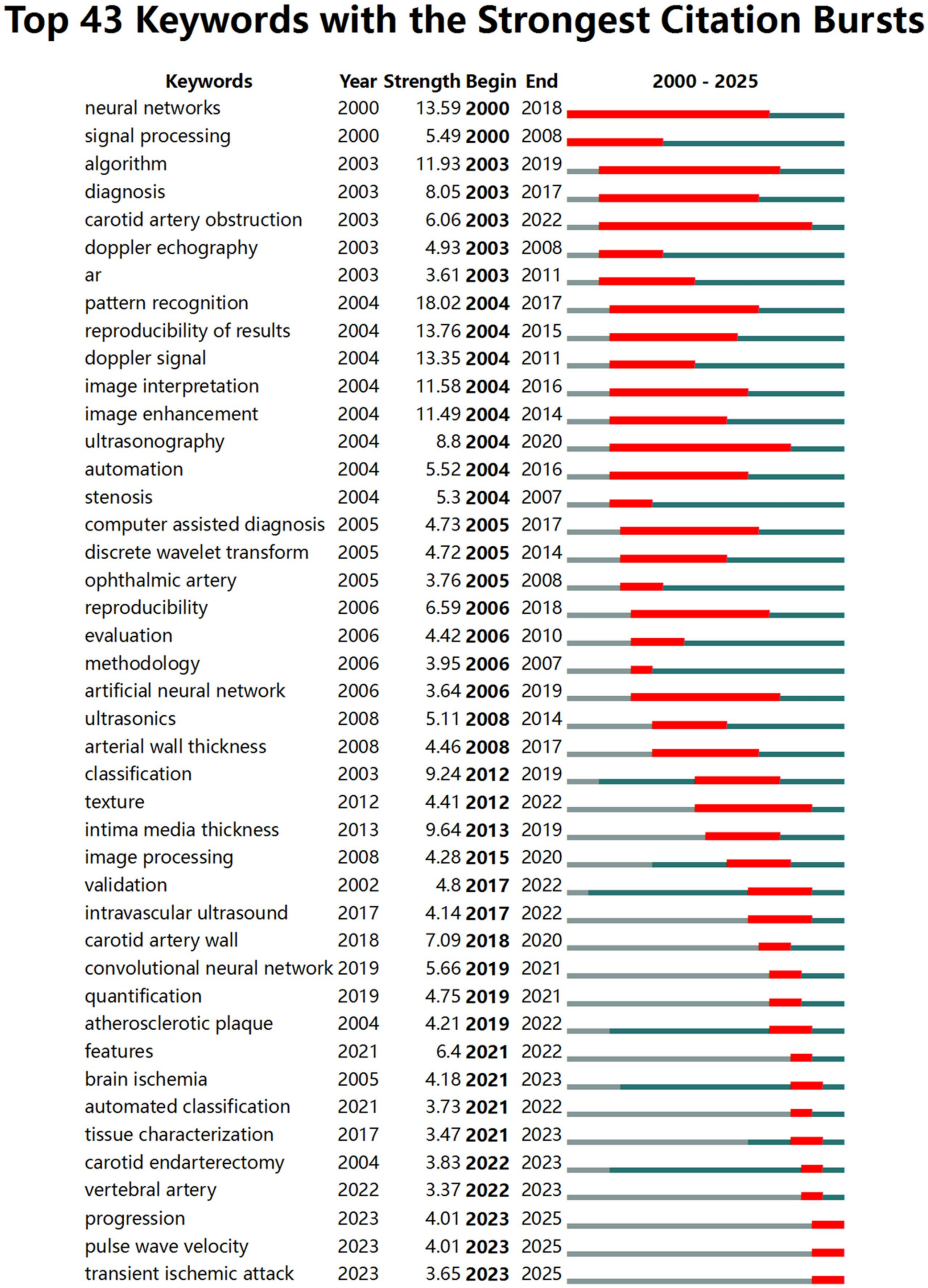
Top 43 keywords with the strongest citation bursts.

CiteSpace published the top 43 keywords of the citation explosion (Figure 5), highlighting emerging research trends and developments. In the early stage, keywords like “signal processing,” “neural network,” and “pattern recognition” dominated, alongside “ultrasonography” and “doppler signal” reflecting a focus on foundational computational methods and ultrasound-based diagnostics. In the subsequent period, bursts of “computer assisted diagnosis”, “classification”, and “reproducibility” indicate a shift toward standardized, automated frameworks, while disease-specific terms such as “stenosis” and “atherosclerotic plaque” highlight growing clinical relevance. More recently, keywords including “CNN”, “tissue characterization”, “IMT”, and “quantification” point to the adoption of deep learning and quantitative imaging, with clinically outcome-oriented keywords like “acute ischemic stroke” and “transient ischemic attack” emphasizing risk assessment and disease progression. By simply dividing the periods of keyword bursts, the study reveals that carotid artery disease research has gradually evolved from basic signal processing to precise assessment and prognostic models, which are deeply integrated with clinical applications and focused on AI. This evolution in research focus is consistent with the overall development trend of intelligent healthcare and precision medicine. It clearly demonstrates the field’s trajectory from early signal processing and neural network methods to advanced AI models for vascular disease diagnosis and prognostic assessment. A more detailed discussion of the staging will be presented in the next section.

## 4 Discussion

Using bibliometric methods, we comprehensively and systematically explored and analyzed the global landscape of AI in carotid research from 2000 to 2025. The annual publication output followed an exponential growth trend (*R*^2^ = 0.9009), corresponding to an approximate annual increase of 15% and a doubling time of about 4–5 years. The observed acceleration in publication output over the last decade, particularly in recent years, reflects the rapid maturation of AI methodologies and their increasing relevance to vascular imaging and cerebrovascular risk assessment. Rather than representing incremental growth, this trend suggests a structural transition in which AI has shifted from exploratory computational applications toward clinically oriented and translational research.

Compared with previous reviews that primarily focused on algorithmic performance or specific imaging modalities (Eini et al., 2025; Huang et al., 2023; Wang and Yao, 2023), the present analysis offers a macro-level perspective by integrating publication dynamics, collaborative structures, and thematic evolution. The prominent contributions from China and the United States illustrate complementary development pathways: China demonstrates rapid expansion in research output and institutional participation, while the United States maintains a central role in global collaboration networks and high-impact knowledge dissemination. This pattern is consistent with broader trends in medical AI research and highlights how national research ecosystems shape both productivity and influence within the field. Although country-level comparisons provide insight into global research distribution, the results were not normalized by population size or research funding. Countries with larger scientific infrastructures may therefore produce more publications. Future studies may incorporate normalized indicators to provide a more balanced cross-national comparison.

Most top journals in this field are based in the U.S., including *Circulation*, *JACC*, and *Radiology*, with the UK’s *Lancet* also exerting notable influence. The concentration of influential journals in North America and Europe further underscores the dominance of developed research systems in shaping academic discourse on AI-assisted carotid research. High-impact journals serve not only as dissemination platforms but also as gatekeepers that influence research standards, clinical relevance, and methodological rigor. This concentration may also be influenced by editorial preferences toward methodologically rigorous and clinically impactful studies, as well as established citation networks that reinforce visibility within high-impact journals. While this structure supports the advancement of high-quality research, it also reinforces existing geographical imbalances in scientific visibility and participation. A particularly concerning finding is the pronounced underrepresentation of low- and middle-income regions, despite these areas bearing a disproportionate burden of cardiovascular disease (Luo et al., 2024). The limited research output from such regions likely reflects structural barriers, including restricted access to advanced imaging technologies, computational infrastructure, large-scale clinical datasets, and interdisciplinary research environments, as well as challenges related to regulatory and ethical frameworks. This difference may prevent these regions from benefiting from relevant research and aggravate the imbalance of medical resources (Suri et al., 2022; Porcu et al., 2022). In addition, due to the lack of AI support, underdeveloped regions will also face obstacles in disease research and treatment innovation (Wang and Zhu, 2026). Therefore, we strongly advocate that global researchers pay greater attention to underdeveloped regions and provide technical support through coordinated strategies, including strengthening international research partnerships, improving access to shared imaging and clinical datasets, and supporting infrastructure development and training programs in resource-limited settings, so as to reduce the health burden and socioeconomic consequences of carotid artery disease.

Consistent with the distribution of leading countries and institutions, influential research in this field has largely been driven by a small number of core research groups based in China, North America, and Europe. Notably, a collaborative research group led by Suri, Saba, and Laird has contributed a substantial proportion of publications in this field, focusing on AI-based carotid plaque analysis for cardiovascular risk assessment (Araki et al., 2016; Saba et al., 2022; Saba et al., 2024; Jamthikar et al., 2019). Collectively, their work demonstrates that AI-enhanced imaging analysis can achieve improved predictive performance beyond conventional assessment methods, underscoring the strategic importance of AI in advancing carotid artery research.

A retrospective analysis of the field based on keyword clustering showed that AI-based carotid artery research can be divided into seven main types: 1. Medical image and hemodynamic analysis: (1) Image segmentation and feature extraction: AI-based classification, segmentation and plaque feature extraction of ultrasound, CT and magnetic resonance imaging (MRI images, etc.), accurately identify and measure lesion sites to improve the detection and diagnosis efficiency of carotid artery diseases (such as atherosclerosis, plaques, stenosis, etc.) (Liu et al., 2023; Gui et al., 2023). (2) Radiomics: Using deep learning and machine learning models to extract high-dimensional features from images to build disease prediction and risk assessment models (Chen C. et al., 2023; Buckler et al., 2021). (3) Hemodynamics and simulation analysis: Computational fluid dynamics combined with AI-based algorithms are used to model carotid blood flow, quantify hemodynamic parameters such as arterial wall shear stress and flow velocity, and thereby investigate the influence of blood flow on plaque formation and the risk of plaque rupture (He et al., 2023; Varga-Szemes et al., 2021). 2. Disease prediction and risk assessment: (1) Disease prediction: Based on the patient’s clinical data and imaging data, predict the individual’s risk of cardiovascular disease or other diseases and their progression, providing a basis for prevention and early intervention (Menchón-Lara et al., 2016; Skandha et al., 2020; Bhagawati et al., 2024). (2) Plaque stability and rupture risk assessment: Integrate deep learning with imaging omics and machine learning to assess the stability of carotid plaques and their risk of rupture, and predict relevant clinical outcomes (Biswas et al., 2020; Li Y. et al., 2024; Chen X. et al., 2023). 3. Basic research and mechanism exploration: (1) Biological mechanism of carotid plaque: explore the molecular mechanism of plaque formation, study the composition of plaque (such as lipids, calcification, fibrous tissue, etc.) to understand the dynamic change process of plaque, and reveal the molecular basis of unstable plaque (James et al., 2023). (2) Genetics and phenotypic studies of atherosclerosis: Machine learning approaches integrated with genome-wide association studies are used to identify genetic variants associated with atherosclerosis and plaque formation and to investigate the relationships between genetic susceptibility and the development of atherosclerotic disease (Song et al., 2024; Theofilatos et al., 2023; Adams et al., 2020). 4. Personalized treatment and precision medicine: (1) Recommendation of individualized treatment plan: Based on carotid artery imaging characteristics and complementary clinical data, these approaches support the development and implementation of treatment strategies and facilitate personalized clinical decision-making (Li B. et al., 2024; Sakakura et al., 2024; Miceli et al., 2023). (2) Treatment effect evaluation and follow-up management: Based on risk factor identification, automatic warning of possible health risks (Xu et al., 2024; Amritphale et al., 2021). 5. Mild-burden monitoring and telemedicine: (1) Smart wearable devices: Use AI combined with wearable devices to monitor carotid artery blood flow changes and pathological conditions in real time and non-invasively, and detect abnormalities at an early stage (Aghilinejad et al., 2024). (2) Telemedicine system: AI-integrated telemedicine platforms are being developed to improve patient management by enabling remote consultation, monitoring, and decision support (Lareyre et al., 2022). 6. Multimodal data fusion and big data analysis: This technical architecture accomplishes full-process optimization from data acquisition to clinical decision-making through coordinated multimodal data fusion and big data analytics, establishing an extensible technological infrastructure for carotid artery research (Banchhor et al., 2018; Geroski et al., 2023; Lv et al., 2023). 7. Virtual Simulation and Surgical Simulation: Simulate the physiological and pathological conditions of the carotid artery for virtual surgical training and treatment planning (Qiu et al., 2023).

The thematic structure of AI-assisted carotid artery research, as revealed by keyword clustering, highlights its increasingly clinical orientation. Current studies predominantly focus on image analysis, disease risk prediction, and clinical decision support, reflecting a convergence of methodological development and disease-centered research objectives. Traditional pathological features such as carotid plaque, stenosis, and intima–media thickness remain central research targets, while clinical outcome–related topics, including stroke and cardiovascular risk, highlight the translational relevance of this field. Widely used imaging modalities such as ultrasound, CT angiography, CT, and MRI continue to serve as the primary data sources for AI model development, reinforcing their role in both research and routine clinical practice.

Based on the analysis of keyword burst trend, this field can be divided into four distinct stages: Exploration Stage (2000–2004), Accumulation Stage (2005–2010), Development Stage (2011–2015), and Integration Stage (2016–2025). The Exploration Stage, or Early Stage of Diagnosis Based on Neural Networks and Expert Systems, was characterized by fundamental computational methods and basic pattern recognition applied to medical data and imaging, supporting preliminary diagnostic models. Research during this period primarily validated neural networks and expert systems (Fan et al., 2001; Fan et al., 2004), developing classic architectures and algorithms while optimizing training efficiency, laying the essential technical groundwork for subsequent medical AI developments, with medical applications emerging secondarily (Übeyli and Güler, 2003; Baldassarre et al., 2004; Piliouras et al., 2004; Cullinane et al., 2000; Hodge et al., 2004; Leoncini et al., 2002).

The Accumulation Stage, or “Neural Network–Based Temporal Signal Classification Stage,” was characterized by modest improvements in computing power, although hardware limitations such as memory and processing speed continued to restrict development. Research during this Stage focused on time-series ultrasound signals due to lower computing demands, developing neural networks and classification algorithms for atherosclerosis and exploring AI-based clinical image analysis for plaque detection, and marking the initial exploration of medical image processing that laid the foundation for subsequent image analysis (Stoitsis et al., 2006; Ozbay et al., 2007; Ceylan et al., 2007; Ceylan et al., 2008; Ozşen et al., 2007; Mougiakakou et al., 2007; Kyriacou et al., 2009).

The Development Stage, or Stage of Development of Machine Learning algorithms and Radiomics, was defined by substantial advances in machine learning algorithms and the emergence of radiomics, driven by rapid growth in computational capacity. Deep learning techniques began to penetrate automated medical image analysis, enabling quantitative characterization of carotid artery pathology (Menchón-Lara et al., 2014; Menchón-Lara and Sancho-Gómez, 2015). Radiomics facilitated high-dimensional feature extraction to support disease classification and risk stratification, reflecting a shift toward data-driven modeling (Acharya et al., 2013; Zuluaga et al., 2011). This stage was predominantly characterized by feature-engineering–based machine learning rather than end-to-end deep learning (Acharya et al., 2012; Sharma et al., 2015), as evidenced by burst keywords related to texture analysis, classification tasks, and IMT, establishing a standardized methodological framework that accelerated clinical translation.

The Integration Stage, or AI and Clinical Integrated Application Stage, represents a phase of deep integration between advanced AI techniques and clinical applications. Breakthroughs in deep learning (Tobore et al., 2019; Gibson et al., 2018; Greenspan et al., 2016; Ravi et al., 2017), particularly CNN, substantially improved the automation and accuracy of carotid image analysis, supporting early diagnosis, risk assessment, and clinical decision-making (Kim et al., 2018; Liu et al., 2019; Wu et al., 2019; Zhou et al., 2024). Research during this stage increasingly emphasized clinically oriented integration of AI models, focusing on disease characterization, outcome prediction, and procedural decision support rather than algorithm development alone (Biswas et al., 2024; Cano et al., 2023; Huang et al., 2025; Sakakura et al., 2024; Lee et al., 2024; Li J. et al., 2024). Burst keywords such as “CNN,” “carotid atherosclerotic plaque”, “tissue characterization”, and “carotid endarterectomy” reflect the expansion of AI applications from diagnostic tasks to prognosis evaluation, intervention planning, and personalized management.

From a clinical perspective, these developments underscore the increasingly important role of AI as a supportive tool in carotid artery disease management. Currently, several commercial AI solutions have been integrated into clinical practice to improve diagnostic efficiency and consistency. For instance, Mindray has introduced the Smart ICV system for intelligent carotid assessment, while CHISON provides the SonoAI platform for automated intima-media thickness measurement. Additionally, global industry leaders such as GE HealthCare and Philips have developed AI-driven quantification tools to standardize plaque analysis and vascular assessment. By improving diagnostic consistency, enhancing risk prediction, and enabling personalized treatment strategies, AI-based approaches have the potential to complement clinician expertise and reduce diagnostic variability. With the continuous growth of clinical data and advances in algorithmic techniques, future research is expected to place greater emphasis on the interpretability, robustness, and clinical adaptability of AI models. In particular, multi-task learning frameworks integrating multimodal imaging data, such as CT, MRI, and ultrasound, may become an important research direction, enabling more comprehensive and accurate disease assessment. Moreover, the integration of large-scale clinical data and personalized medicine is likely to further improve the precision and effectiveness of AI-driven predictive models and individualized treatment planning. However, similar to most studies involving AI, carotid artery research based on AI also faces multiple challenges that need to be addressed, including privacy and data security, imbalance and heterogeneity of data quality, lack of interpretability and transparency, cost and resource accessibility. However, similar to most studies involving AI, carotid artery research based on AI also faces multiple challenges that need to be addressed, including privacy and data security, imbalance and heterogeneity of data quality, lack of interpretability and transparency, cost and resource accessibility.

Our study has several limitations that should be acknowledged. First, the analysis relies on the completeness and accuracy of existing literature and may not capture all relevant studies, particularly non-English or non-indexed publications, which may introduce language and database selection biases. Second, researchers’ choices of keywords and subject terms, as well as the exclusion of rare or uncommon keywords, may have narrowed the scope of analysis and led to the omission of certain publications. In addition, citation-based indicators were not normalized by publication year, meaning earlier publications may have had more time to accumulate citations than recently published studies, which may introduce citation bias. Furthermore, while bibliometric analysis provides a macro-level quantitative overview of research trends and identifies influential contributors, it possesses inherent constraints regarding clinical interpretation. As noted in recent literature, bibliometrics excels at mapping research hotspots, yet it often prioritizes citation impact over the immediate clinical utility or granular technical robustness of specific findings (Chou et al., 2026). Moreover, inherent constraints of bibliometric tools such as CiteSpace, including potential inaccuracies in keyword clustering, citation parsing, and visualization of trends, may affect the interpretation of results. Therefore, the findings should be considered a general overview of research trajectories rather than an exhaustive or qualitatively-validated representation of AI-based carotid research.

In summary, through a comprehensive analysis of the literature in the field of AI carotid research, we have identified the development trajectory and pattern of this field in the past 25 years, demonstrating the importance and potential of AI for carotid research. Future research should leverage the advantages of AI carotid research, focus on the interpretability, clinical applicability, big data, precision medicine, multi-omics, and multimodality of AI models, address the challenges faced in current research, and more fully translate these research results into clinical applications. In addition, while recognizing the significant contributions of researchers and institutions from North America, Europe, and East Asia, we hope that the phenomenon of geographical imbalance in research can attract sufficient attention. Researchers and policymakers need to face these differences to improve the imbalance in the allocation of medical resources and promote the development of the medical industry.

## Data Availability

The raw data supporting the conclusions of this article will be made available by the authors, without undue reservation.
