# Supplementary material for: Artificial intelligence in carotid research: a 25-year bibliometric analysis of global trends and future directions
Source: Front Artif Intell. 2026 May 19;9:1716935. doi: 10.3389/frai.2026.1716935 (PMC13226204; doi:10.3389/frai.2026.1716935)
Supplement: Supplementary file 1 [file Data_Sheet_1.DOCX]

Supplementary Material S1. Detailed Search Strategies for Each Database

WoSCC:

(TS=("Machine Learning" OR "Artificial Intelligence" OR "Deep learning" OR "Supervised model*" OR "Unsupervised model*" OR "Supervised algorithm*" OR "Unsupervised algorithm*" OR "supervised learning" OR "Unsupervised learning" OR "Semi-supervised learning" OR "Reinforcement learning" OR "AI algorithm*" OR "DL algorithm*" OR "ML algorithm*" OR "AI model*" OR "DL model*" OR "ML model*" OR "neural network*" OR "Large Language Model*" OR "Generative Pre-Trained Transformer" OR "Radiomic*" OR "Ultrasomic*") )AND (TS=("Carotid Arter*" OR "Caroti*" OR "neck arter*")) NOT (TS=(animal OR mouse OR mice OR rat OR rats OR rabbit* OR dog* OR pig* OR swine* OR cow* OR cattle* OR Sheep* OR goat* OR murine*))

Filters: Limite to Article, English, from 2000 – 2025

Scopus:

( KEY ( "machine learning" OR "artificial intelligence" OR "deep learning" OR "supervised learning" OR "unsupervised learning" OR "semi-supervised learning" OR "reinforcement learning" OR "neural network" OR "neural networks" OR "large language model" OR "generative ai" OR "radiomic" OR "radiomics" OR "ultrasomic" OR "ultrasomics" ) OR TITLE-ABS ( "supervised model*" OR "unsupervised model*" OR "supervised algorithm*" OR "unsupervised algorithm*" OR "ai algorithm*" OR "dl algorithm*" OR "ml algorithm*" OR "ai model*" OR "dl model*" OR "ml model*" OR "transformer model" OR "gpt" ) ) AND ( KEY ( "carotid artery" OR "carotid arteries" ) OR TITLE-ABS ( "carotid arter*" OR "caroti*" OR "neck arter*" ) ) AND NOT ( TITLE-ABS-KEY ( animal OR mouse OR mice OR rat OR rats OR rabbit* OR dog* OR pig OR swine OR cow OR cattle OR Sheep* OR goat* OR murine* ) ) AND PUBYEAR > 1999 AND PUBYEAR < 2026 AND ( LIMIT-TO ( DOCTYPE , "ar" ) ) AND ( LIMIT-TO ( LANGUAGE , "English" ) )

PubMed：

(((("Machine Learning"[Title/Abstract] OR "Artificial Intelligence"[Title/Abstract] OR "deep learning"[Title/Abstract] OR "supervised model*"[Title/Abstract] OR "unsupervised model*"[Title/Abstract] OR "supervised algorithm*"[Title/Abstract] OR "unsupervised algorithm*"[Title/Abstract] OR "supervised learning"[Title/Abstract] OR "Unsupervised learning"[Title/Abstract] OR "Semi-supervised learning"[Title/Abstract] OR "Reinforcement learning"[Title/Abstract] OR "ai algorithm*"[Title/Abstract] OR "dl algorithm*"[Title/Abstract] OR "ml algorithm*"[Title/Abstract] OR "ai model*"[Title/Abstract] OR "dl model*"[Title/Abstract] OR "ml model*"[Title/Abstract] OR "neural network*"[Title/Abstract] OR "large language model*"[Title/Abstract] OR "Generative Pre-Trained Transformer"[Title/Abstract] OR "radiomic*"[Title/Abstract] OR "ultrasomic*"[Title/Abstract] OR ("Machine Learning"[MeSH Terms] OR "Artificial Intelligence"[MeSH Terms] OR "neural networks, computer"[MeSH Terms] OR "deep learning"[MeSH Terms])) AND ("carotid arter*"[Title/Abstract] OR "caroti*"[Title/Abstract] OR "neck arter*"[Title/Abstract] OR "Carotid Arteries"[MeSH Terms])) NOT ("Animals"[MeSH Terms] NOT "Humans"[MeSH Terms])) AND ("journal article"[Publication Type] NOT ("review"[Publication Type] OR "systematic review"[Publication Type] OR "meta-analysis"[Publication Type]))) AND ((english[Filter]) AND (2000:2025[pdat]))
